# Enhanced antitumor efficacy of cisplatin in combination with HemoHIM in tumor-bearing mice

**DOI:** 10.1186/1471-2407-9-85

**Published:** 2009-03-17

**Authors:** Hae-Ran Park, Eun-Jin Ju, Sung-Kee Jo, Uhee Jung, Sung-Ho Kim, Sung-Tae Yee

**Affiliations:** 1 Radiation Resarch Division for Bio-Technology, Advanced Radiation Technology Institute, Jeongeup Campus of Korea Atomic Energy Research Institute (KAERI), 1266 Sinjeong-dong Jeongeup-si Jeonbuk 580-185, Republic of Korea; 2 College of Veterinary Medicine, Chonnam National University, Gwangju 500-757, Republic of Korea; 3 Department of Biology, Sunchon National University, Sunchon 540-742, Republic of Korea

## Abstract

**Background:**

Although cisplatin is one of the most effective chemotherapeutic agents, cisplatin alone does not achieve a satisfactory therapeutic outcome. Also cisplatin accumulation shows toxicity to normal tissues. In this study, we examined the possibility of HemoHIM both to enhance anticancer effect with cisplatin and to reduce the side effects of cisplatin in melanoma-bearing mice.

**Methods:**

HemoHIM was prepared by adding the ethanol-insoluble fraction to the total water extract of a mixture of 3 edible herbs, Angelica Radix, Cnidium Rhizoma and Paeonia Radix. Anticancer effects of HemoHIM with cisplatin were evaluated in melanoma-bearing mice. We used a Cr^51^-release assay to measure the activity of NK/Tc cell and ELISA to evaluate the production of cytokines.

**Results:**

In melanoma-bearing mice, cisplatin (4 mg/kg B.W.) reduced the size and weight of the solid tumors, and HemoHIM supplementation with cisplatin enhanced the decrease of both the tumor size (p < 0.1) and weight (p < 0.1). HemoHIM itself did not inhibit melanoma cell growth *in vitro*, and did not disturb the effects of cisplatin *in vitro*. However HemoHIM administration enhanced both NK cell and Tc cell activity in mice. Interestingly, HemoHIM increased the proportion of NK cells in the spleen. In melanoma-bearing mice treated with cisplatin, HemoHIM administration also increased the activity of NK cells and Tc cells and the IL-2 and IFN-γ secretion from splenocytes, which seemed to contribute to the enhanced efficacy of cisplatin by HemoHIM. Also, HemoHIM reduced nephrotoxicity as seen by tubular cell of kidney destruction.

**Conclusion:**

HemoHIM may be a beneficial supplement during cisplatin chemotherapy for enhancing the anti-tumor efficacy and reducing the toxicity of cisplatin.

## Background

Chemotherapy has been one of the major therapeutic modalities commonly used for the treatment for a variety of cancer patients. However, in many cases, chemotherapy or radiotherapy alone cannot achieve a satisfactory therapeutic outcome, namely the complete remission of tumors, and induces severe side effects at therapeutically effective doses.

Cisplatin (cis-diaminedichloroplatinum (II) or CDDP), a platinum-containing anticancer drug, is one of the most commonly used cytotoxic agents for the treatment of a variety of solid malignant tumors. Despite its excellent anticancer activity, the clinical use of cisplatin is often limited by its undesirable side effects, such as severe nephrotoxicity and hepatotoxicity [1,2]. Although the precise mechanism for the cisplatin-induced toxicity is not well understood, cisplatin is preferentially taken up and accumulated in the liver and kidney cells [3], resulting in the enhanced production of reactive oxygen species (ROS) and the decrease in antioxidant enzymes [4-7]. Therefore, antioxidants have been administered before cisplatin treatment to protect against nephrotoxicity [8-10].

Complementary and alternative medicine defined by the National Center as a group of diverse medical and health care systems is not normally considered to be conventional medicine [11]. Complementary and alternative medicine does not inhibit tumor growth. These treatments might be undertaken adjuvant to, or instead of, conventional treatments. Numerous herbal medical products are promoted as complementary and alternative medicines. In addition, scientific and medical studies in Korea, China, and Japan, and more recently in the United States, have increasingly shown that plant-derived polysaccharides have potent immunotherapeutic properties with respect to the prevention and treatment of cancer [12-19].

A new herbal composition, HemoHIM, was designed by adding its polysaccharide fraction into a hot water extract of an herb mixture consisting of Angelica Radix, Cnidium Rhizoma and Paeonia Radix. This composition was designed to protect the self-renewal tissues and to promote a recovery of the immune system against oxidative stresses, such as irradiation [20,21]. The general composition of HemoHIM were 60.4% carbohydrate, 6% protein and 33.6% other (Data not shown). The immune modulating components in HemoHIM were the ethanol-insoluble fraction [21], and the polysaccharide content in this fraction was 40.9% (± 3.8) (Data not shown). In addition, the functional components included in the ethanol-soluble fraction of HemoHIM were Gallic acid [0.2% (± 0.06)], chlorogenic acid [0.33% (± 0.05)], paeoniflorin [1.32% (± 0.15)], nodakenin [0.58% (± 0.04)] and benzoic acid [0.17% (± 0.05)] (Data not shown). Especially, these 3 herbs are listed as raw materials in the Korea Food Code. Finally, HemoHIM has been proven to be safe for long-term administration (data not shown).

Surveillance capacity to tumor cells was mediated nonspecifically by dendritic cells, macrophages and natural killer cells and was mediated specifically by T cells including cytotoxic T (Tc) cells. Both T and NK cells have been shown to be anticancer effector cells [22,23]. Also, IFN-γ and IL-2 have been shown to have anticancer activity in animals [24,25].

In this study, the possibility of HemoHIM increasing the immune surveillance capacity to tumor cells through both NK cells and Tc cells in melanoma-bearing mice treated with cisplatin was assessed. Also, we examined its protective effect against cisplatin-induced nephrotoxicity in melanoma-bearing mice. Our results suggest that HemoHIM may be a beneficial supplement during cisplatin chemotherapy by enhancing the anti-tumor efficacy and reducing the toxicity of cisplatin.

## Methods

### Animals

Research was approved and conducted according to the principles enunciated in the 'Animal Care Act', prepared by the Ministry of Agriculture and Forestry, Republic of Korea. 8-week-old female C57BL/6 (H-2^b^) mice were used (The Orient Inc.; Charles River Technology; Seoul, Korea). The mice were housed in polycarbonate cages under a specific pathogen-free condition, and were fed with a standard animal diet and water *ad libitum*.

### Preparation of HemoHIM

A mixture of 3 edible medicinal herbs, Angelica Radix (root of *Angelica gigas *Nakai), Cnidii Rhizoma (rhizome of *Cnidium officinale *Makino), and Paeonia Radix (root of *Paeonia japonica *Miyabe), was decocted for 4 hours in boiling water to obtain a total extract (HIM-I). One half of HIM-I was fractionated into an ethanol-soluble fraction and into an ethanol-insoluble polysaccharide fraction by a precipitation in 80% ethanol. HemoHIM was prepared by adding the ethanol-insoluble polysaccharide fraction to the other half of HIM-I.

### Cell culture

B16F0 (Melanoma cell line; CRL-6322) and YAC-1 (Molony virus induced leukemia; TIB-160) were purchased from ATCC (Rockville, MD, USA) and cultured in RPMI 1640 supplemented with 10% fetal bovine serum (FBS, GIBCO BRL, Grand Island, USA)), 2 × 10^-2 ^M HEPES buffer, 2 × 10^-3 ^M L-glutamine, 100 U/ml penicillin and 50 μg/ml streptomycin (GIBCO BRL). All cells were grown at 37°C in a humidified atmosphere containing 5% CO_2_.

### Cisplatin injection and HemoHIM administration in tumor-bearing mice model

**Figure 1:**
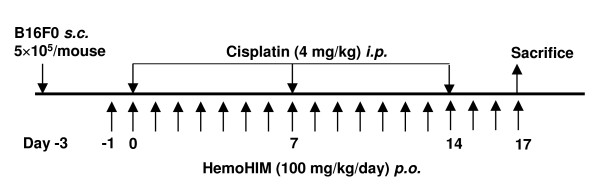
**The experimental schedule used for assessing the efficacy of HemoHIM in melanoma-bearing mice which were injected with cisplatin**. B16F0 melanoma cells (5 × 10^5^/mouse) were inoculated into subcutaneous femoral left region of mice 3 days before an initial injection of cisplatin. Cisplatin was intraperitoneally injected at 4 mg/kg B.W., and HemoHIM was daily given at 100 mg/kg B.W. from day -1 to day 16. On day 17 after initial injection of cisplatin, all mice of each group were experimented, respectively, to evaluate various parameters.

Mice were divided randomly into three groups (Control, Cisplatin and Cisplatin+HemoHIM), and each group consisted of twenty mice. B16F0 melanoma (5 × 10^5 ^cells/mouse) was inoculated into subcutaneous femoral left region of mice at 3 days before an initial injection of cisplatin. Cisplatin was injected intraperitoneally at 4 mg/kg body weight (B.W.) on day 0, 7 and 14 (total three injections). Experimental group was intubated with HemoHIM at a final concentration of 100 mg/kgB.W. by everyday from day -1 to day 16, while the control group received only water. The scheme of the administration procedure is summarized in Fig. 1. On day 17 after initial injection of cisplatin, all mice of each group were experimented, respectively, to evaluate tumor weight or tumor size. The tumor size was calculated as follows: tumor size = ab^2^/2, where a and b are the larger and smaller diameters, respectively.

### Melanoma cell growth assay *in vitro*

The effect of cisplatin and HemoHIM on the number of viable melanoma cells was assessed by CCK-8 (WST-8; Dojindo Lab, Kumamoto, Japan). The principle underlying the cell viability assessment by CCK-8 is based on the dehydrogenase activity detection in viable cells, similar with the principle of MTT. Briefly, the melanoma cells were seeded on to 96-well plates at a density of 7 × 10^3 ^cells per well in 100 μl of media. After incubation for 24 hours, cells were treated with various concentration of cisplatin and with HemoHIM 100 μg/ml for 24 hours. After incubation, a CCK-8 solution was added to each well. Cells were incubated at 37°C for two hours and the optical density was measured using microplate reader (Molecular Devices) at 450 nm (-Ref. 570 nm).

### Preparation of lymphocyte in the spleen

Spleens were removed aseptically from the mice and a single cell suspension was prepared by mincing the spleen. The spleen lymphocytes were prepared by a density gradient centrifugation on a Ficoll-Hypaque solution (Sigma-Aldrich Co., St. Louis, MO, USA). All the cell suspensions were maintained in the RPMI 1640 media supplemented with 10% fetal bovine serum (FBS), 2 × 10^-2 ^M HEPES buffer, 2 × 10^-3 ^M L-glutamine, 1 × 10^-3 ^M pyruvate, 100 U/ml penicillin, 50 μg/ml streptomycin, 5 × 10^-5 ^M of 2-mercaptoethanol and 1% nonessential amino acid. All the supplements were purchased from GIBCO BRL (Grand Island, USA).

### HemoHIM administration in MMC-treated tumor-bearing mice model

Firstly, harvested B16F0 melanoma was inactivated by mitomicin C (MMC) treatment (50 μg/ml, incubation for 40 minutes at 37°C water bath). MMC-treated B16F0 melanoma were inoculated intraperitoneally with 1 × 10^6 ^cells/mouse 3 days after an initial administration of HemoHIM. Experimental group was intubated with HemoHIM at a final concentration of 100 mg/kgB.W. by everyday from day -3 to day 4, while the control group was received only water. Ten days after cancer cell inoculation, spleen lymphocytes were prepared as effector cells to measure the activity of NK cells and Tc cells through Cr^51^-release assay.

### Assay for NK cell and cytotoxic T (Tc) cell mediated cancer cell killing activity

YAC-1 tumor targets or B16F0 tumor targets were labeled with ^51^Cr-sodium chromate (Amersham Pharmacia Biotech, Kangnam-ku, Seoul, Korea) at a dose of 40 μCi/10^6 ^cells for 60 min to measured NK cell activity or Tc cell activity, respectively. The cells were washed three times in HBSS (Hank's Balanced Salt Solution; Sigma-Aldrich Co.) and resuspended to a final concentration of 2 × 10^5 ^cells/ml. Twenty thousand target cells and 10^6 ^or 2 × 10^6 ^spleen effector cells were plated into the wells of a 96-well U bottom plate. The plates were then incubated at 37°C for 4 hr in humidified air containing 5% CO_2_. Following a centrifugation at 350 g for 10 min, 100 μl of the supernatant was harvested from each well and counted for 1 min in a gamma counter (Wallac, Wellesley, MA, USA). The percent lysis was calculated as follows: % lysis = {[CPM (experimental) - CPM (spontaneous)]/[CPM (maximum) - CPM (sponstaneous)]} × 100.

### Flow cytometry analysis of NK and Tc cells in spleen lymphocytes

The spleen lymphocytes were stained with fluorescent-labeled antibodies or isotype control antibodies in phosphate buffered saline (PBS). After staining for 30 minutes, the lymphocytes were washed three times with a fresh FACS media and then analyzed by flow cytometry (Backman Coulter, Miami, Florida, USA). A fluorescence histogram of at least 50,000 counts was analyzed in each sample. The following reagents from BD PharMingen (San Diego, CA, USA) were used: PE-conjugated anti-NK1.1 and FITC-conjugated anti-CD8.

### Condition for a cytokine production *in vitro*

The spleen lymphocytes (2 × 10^6 ^cells/well) obtained from the mice were stimulated with concanavalin A (ConA) at 1 μg/ml for 1 or 2 days to measure IL-2 and IFN-γ level in supernatant.

### Antibodies and the enzyme-linked immunosorbent assay (ELISA)

For the IL-2, clone JES6-1A12 was used as the capture Ab, and biotin-labelled JES6-5H4 was the detecting Ab. For the IFN-γ measurements, clone R4-6A2 was used as the capture Ab, and biotin-labeled XMG1.2 was the detecting Ab. All the antibodies as well as the recombinant IFN-γ and IL-2 were purchased from BD PharMingen (San Diego, CA, USA). Cytokines were determined by previously described ELISA method [26].

### Histopathological examination of kidney by hematoxylin and eosin staining

Kidney from mice of each group on day 17 after initial injection of cisplatin was removed and fixed in 10% buffered formalin for 2 days. The paraffin-embedded sections (5 μm thick) were stained with hematoxylin and eosin (H&E) for histolpathological examination and observed under light microscope at × 200 magnifications.

### Statistical analysis

Data were expressed as mean ± S.D. and a statistical significance was analyzed by using a Student's *t*-test. Differences with a *p *value of less than 0.05 were taken as significant, and considerable with a *p *value of less than 0.1.

## Results

### HemoHIM enhances the antitumor efficacy of cisplatin in tumor-bearing mice

**Figure 2:**
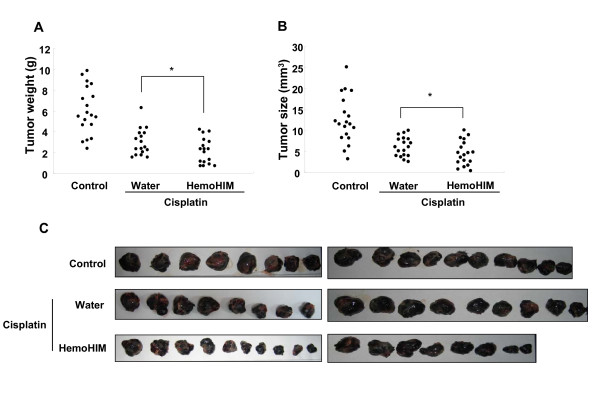
**The inhibition of tumor growth was enhanced by HemoHIM administration in melanoma-bearing mice which were injected with cisplatin**. All mice performed as described in figure 3 were sacrificed at 17 days after initial injection of cisplatin. Tumor weight **(A) **and size **(B) **were measured. **(C) **Photographs of melanoma solid tumor taken from all mice of each group. There were twenty mice in each group. Data show the Mean ± SD. *p < 0.1 compared with only cisplatin treated group.

To assess the effect of HemoHIM on tumor growth inhibition in cisplatin treated B16F0 melanoma-bearing mice, we used a tumor-bearing mice model that was summarized in Fig. 1. As shown in Fig. 2A and 2B, at 20 days after melanoma inoculation, the tumor weight and size of the control group without cisplatin and HemoHIM treatment were 6.093 g (± 2.28) and 12.6 mm^3 ^(± 5.65), respectively. However, in the cisplatin-injected group, tumor weight (2.97 g (± 1.29)) and size (6.3 mm^3 ^(± 2.32)) were reduced significantly in comparison to the control group. HemoHIM supplementation with cisplatin resulted in a further reduction in both the tumor weight (2.22 g (± 1.24)) and size (4.7 mm^3 ^(± 2.93)). Only HemoHIM supplementation without cisplatin showed no reduction in tumor weight [6.15 g (± 1.238)] when compared with the control group (Data not shown). This suggests that HemoHIM itself did not show a reduction effect of tumor growth. Photographs are shown in Fig. 2C.

### HemoHIM enhances the activity of NK and Tc cells rather than directly killing cancer cells

**Figure 3:**
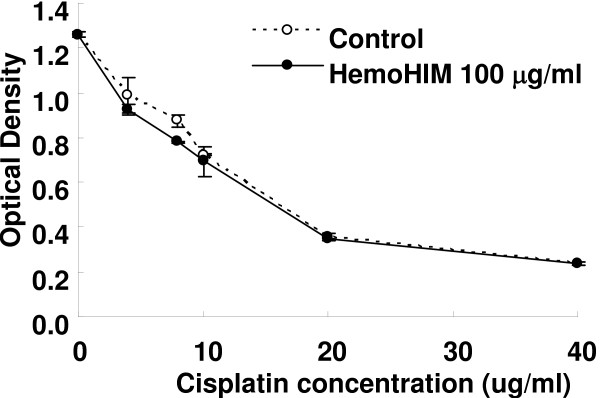
**Growth inhibition effect of cisplatin and HemoHIM on melanoma cells *in vitro***. The melanoma cells were seeded at a density of 7 × 10^3 ^cells per well. After incubation for 24 hours, cells were treated with various concentration of cisplatin and with HemoHIM 100 μg/ml for 24 hours. After incubation, a CCK-8 solution was added to each well for 1 hour and then the optical density was measured.

The anticancer effect of cisplatin mainly depends on its DNA-damaging activity, via its direct interaction with DNA to form DNA adducts [27]. As shown in Fig. 3, cisplatin inhibited melanoma cell growth in a dose-dependent manner, with IC_50 _at about 15 μg/ml. However, HemoHIM itself did not inhibit melanoma growth *in vitro *(Data not shown), nor did it disturb the working of cisplatin *in vitro *(Fig. 3).

**Figure 4:**
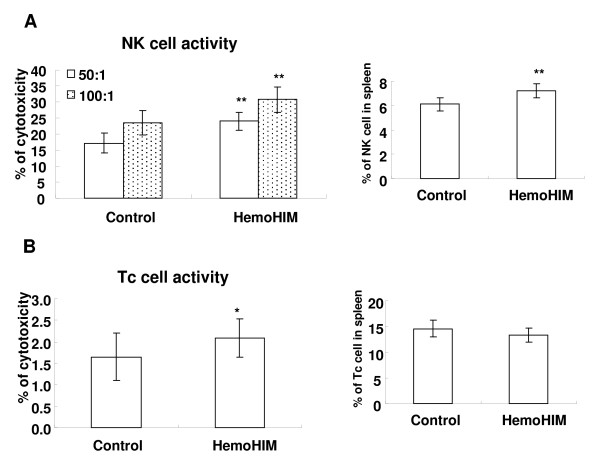
**Effect of HemoHIM on cancer cell killing activity of NK cells and Tc cells**. C57BL/6 mice were orally administrated with HemoHIM (100 mg/kg B.W.) and injected with MMC-treated B16F0 melanoma (1 × 10^6^cell/mouse) into peritoneal cavity. 10 days after cancer cell inoculation, the cancer cell killing activity of NK cells or Tc cells was determined by ^51^Cr release assay as described in *Materials and Methods*. There were six mice in each group. Data show the Mean ± SD. * p = 0.06 and ** p < 0.05 compared with mice administrated water.

Because HemoHIM did not directly kill cancer cells, we thought that the activity of immune cells which were in charge of tumor surveillance may be enhanced by HemoHIM. We investigated the cancer cell-killing activity of NK and Tc cells, as they are in charge of innate and adaptive immunity against tumor, respectively. To investigate the activity of Tc cell, we used the mice which were immunized with mitomycin C (MMC)-treated melanoma cells. As shown in Fig. 4A and 4B, HemoHIM administration enhanced cancer cell-killing activity of NK cells and Tc cells in MMC-treated melanoma cell-bearing mice (p < 0.05 and p = 0.06, respectively). Also, the proportion of NK cell in spleen lymphocytes was increased by HemoHIM administration (p < 0.05), but not the proportion of Tc cells. Specially, these results were important because NK cells take part in both innate and adaptive immunity, and are regarded as interfaces between the innate and adaptive immune systems [28].

### The enhanced efficacy of cisplatin with HemoHIM in melanoma-bearing mice was due to increasing IL-2 and IFN-γ secretion and enhancing the activity of NK and Tc cells

**Figure 5:**
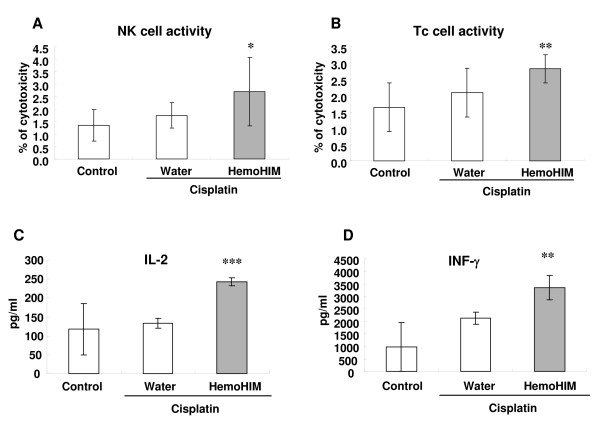
**HemoHIM administration promotes immune responses for tumor surveillance in melanoma-bearing mice which were injected with cisplatin**. All mice performed as described in figure 3 were sacrificed at 17 days after initial injection of cisplatin. **(A)(B) **The cancer cell killing activity of NK cells or Tc cells was determined by ^51^Cr release assay as described in *Materials and Methods*. **(C)(D) **Spleen lymphocytes were cultured with ConA (1 μg/ml). After 1 or 2 days, IL-2 and IFN-γ in culture supernatants were measured by ELISA as described in *Materials and Methods*. There were twenty one mice in each group. The spleens of three mice were pooled. Bars show the means ± SD of the septuple experiments. * p = 0.1, **p < 0.05 and ***p < 0.001 compared with only cisplatin treated group.

As the mechanisms for an enhanced anticancer efficacy of cisplatin in combination with HemoHIM in melanoma cell-bearing mice (Fig. 1), we concentrated on the activity of NK and Tc cells because these cells play an important role on cancer surveillance. As shown Fig. 5A and 5B, cisplatin injection alone in melanoma-bearing mice did not enhance or decrease the activity of NK cells and Tc cells. However, HemoHIM administration enhanced the activity of NK cells in melanoma-bearing mice which were treated with cisplatin (p = 0.1; Fig. 5A). In addition, the activity of Tc cells was enhanced significantly by HemoHIM administration (p < 0.05; Fig. 5B). These data suggested that the enhanced anticancer efficacy of cisplatin in combination with HemoHIM administration was due to the increase in the activity of NK and Tc cells that are in charge of tumor surveillance.

Macrophages, dendritic cells, NK cells and Tc cells that are able to recognize, bind and subsequently kill tumor cells, were activated by cytokines such as IL-2 and IFN-γ. In our previous study, lymphocytes treated with HemoHIM alone enhanced the expression of IL-2 and IFN-γ *in vitro *(data not shown). Truly, IL-2 and IFN-γ are potent activators of NK cell and Tc cell effector functions. We therefore ascertained whether HemoHIM administration enhanced the production of IL-2 and IFN-γ in melanoma-bearing mice treated with cisplatin. HemoHIM administration significantly enhanced IL-2 (p < 0.001; Fig. 5C) and IFN-γ (p < 0.05; Fig. 5D) production in melanoma-bearing mice treated with cisplatin.

### HemoHIM decreases the cisplatin-induced nephrotoxicity in tumor-bearing mice

**Figure 6:**
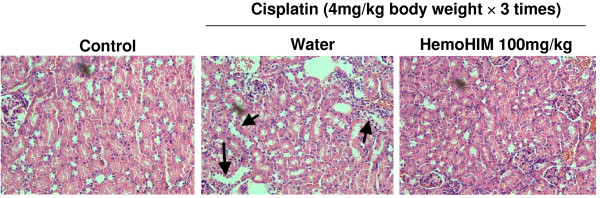
**HemoHIM reduces the cisplatin-induce damage of kidney**. Kidney from mice of each group on day 17 after initial injection of cisplatin was removed and fixed in 10% buffered formalin for 2 days. The paraffin-embedded sections (5 μm thick) were stained with hematoxylin and eosin (H&E) for histolpathological examination and observed under light microscope at × 200 magnifications. The result shown here is representative cortex from each group. **(A) **Control, cortex (× 200), **(B) **Cisplatin control, cortex (× 200), renal tubule cell destruction (marked by arrows), **(C) **Cisplatin+HemoHIM, cortex (× 200).

Undesirable side effects of cisplatin appear in the kidney and liver, due to cisplatin accumulation in these organs [3]. As one of the mechanisms for cisplatin-induced toxicity is the enhanced production of ROS in these organs, we thought that HemoHIM may have a radical scavenging activity [20,21], reducing cisplatin-induced damage. Experimental studies in animals have shown that a minimum dose of cisplatin (5 mg/kg body weight, i.p.) was sufficient to induce nephrotoxicity in rats [29,30]. In this study, the cisplatin was injected at 4 mg/kg body weight three times once weekly. The nephrotoxicity was assessed using kidneys removed from mice on day 17 after an initial injection of cisplatin, and the representative result of each group is shown in Fig. 6. In the histopathological examination of the kidney, cisplatin destroyed renal tubular cells (Fig. 6). However HemoHIM administration reduced the destruction of renal tubular cells by cisplatin.

## Discussion

HemoHIM is an herbal composition designed to protect the self-renewal tissues and to promote the recovery of the immune system against an oxidative stresses such as an irradiation. In this study, we examined the possibility of HemoHIM both to enhance the anticancer effect of cisplatin and to reduce its side effects in melanoma-bearing mice. In our previous studies, HemoHIM was tested for its efficacy as a radioprotective agent [20,21]. Also, we investigated the effect of HemoHIM on a restoration of the immune functions which had been impaired in aged mice [31]. Besides, in our previous study, we showed that HemoHIM accelerated the recovery of immune cells in mice treated with cyclophosphamide, which is a well-known anticancer agent [32]. In this study, unlike cisplatin, HemoHIM alone did not directly kill the cancer cells. Nevertheless, our results showed that HemoHIM administration enhanced antitumor efficacy of cisplatin in melanoma-bearing mice.

Immune responses that are capable of killing tumor cells consist of dendritic cells, macrophages, NK cells and Tc cells. Tc cells may perform a surveillance function by recognizing and killing potentially malignant cells that express peptides derived from mutant cellular proteins or oncogenic viral proteins and presented in association with class I MHC molecules. NK cells kill many types of tumor cells, especially cells that have reduced class I MHC expression and can escape killing by Tc cells [33]. *In vitro *studies using cells from humans and several other mammalian species, as well as *in vivo *studies in mice and rats, have long suggested that tumor cells are recognized as NK cell targets [34]. Also, NK cells act as regulatory cells to influence various other cell types, such as dendritic cells, helper T (Th) cells, Tc cells and B cells [35]. Many studies for immuno-cancer therapy were focused on enhancing the activity of NK cells as well as Tc cells against tumor cells. In our data, cisplatin alone decreased tumor size, but did not enhance the activity of NK cells and Tc cells (Fig. 5). Also, HemoHIM alone without cisplatin did not enhance the activity of these cells (data not shown). However, HemoHIM administration with cisplatin injection increases the activity of NK cells and Tc cells in melanoma-bearing mice (Fig. 5A and 5B), while the tumor size was decreased by cisplatin. Thus, we suggest that the mechanism of action of cisplatin and HemoHIM in tumor-bearing mice differ.

IL-2 and IFN-γ are potent activators of NK cell and Tc cell effecter functions. For these reasons, cytokine therapies of malignant tumors using IL-2, IL-12, and IFN-γ have been investigated extensively in experimental and clinical studies [34]. It is also well known that IL-2 and IFN-γ promote tumor-reactive lymphocyte proliferation, cytotoxicity and, to some extent, cytokine secretion [36-41]. IFN-γ is a pleiotropic immunoregulatory cytokine that has been used for clinical treatment of certain tumors [42,43]. However, the clinical application of such cytokines has been hampered largely due to their undesirable side effects [44,45]. In our data, HemoHIM administration with cisplatin injection increased the secretion of IL-2 and IFN-γ in melanoma-bearing mice.

Although cisplatin is an anticancer drug highly effective against several cancers, cisplatin is toxic to liver and kidney cells through producing ROS [1-4,30]. However, many studies to counter nephrotoxicity through the administration of antioxidants have been performed [8-10]. As HemoHIM was designed to protect the self-renewal tissues and to promote a recovery of the immune system against oxidative stresses [20,21], we thought it may be able to decrease cisplatin-induced kidney damage. As expected, HemoHIM administration reduced nephrotoxicity as seen by tubular cell destruction of kidney.

Based on the overall these results, the possibility of HemoHIM both to enhance anticancer effect of cisplatin and to reduce its side effects in melanoma-bearing mice were ascertained. However, the protective efficacy of HemoHIM on cisplatin-induce damage in the kidney needs to be investigated further.

## Conclusion

In conclusion, although the manner in which HemoHIM administration decreases cisplatin-induced kidney damage remains unknown, our results indicate that HemoHIM may be a useful complementary agent during cisplatin chemotherapy by enhancing the anti-tumor efficacy and reducing the toxicity of cisplatin.

## Competing interests

The authors declare that they have no competing interests.

## Authors' contributions

HRP carried out making HemoHIM, the cell culture, Cr^51^-relaeas assay, animal experiments, and draft out the manuscript. EJJ performed the cell culture, ELISA analysis and histopathological examination. UHJ, SHK and STY participated in the design of study. SKJ conceived of the study, and participated in its design. All authors have read and approved the manuscript.

## Pre-publication history

The pre-publication history for this paper can be accessed here:

http://www.biomedcentral.com/1471-2407/9/85/prepub
